# Are Reduced Levels of Coagulation Proteins Upon Admission Linked to COVID-19 Severity and Mortality?

**DOI:** 10.3389/fmed.2021.718053

**Published:** 2021-09-30

**Authors:** Francisco C. Ceballos, Pablo Ryan, Rafael Blancas, María Martin-Vicente, Erick Joan Vidal-Alcántara, Felipe Peréz-García, Sofía Bartolomé, Juan Churruca-Sarasqueta, Ana Virseda-Berdices, Oscar Martínez-González, Oscar Brochado-Kith, Marta Rava, Carolina Vilches-Medkouri, Natalia Blanca-López, Ignacio Ramirez Martinez-Acitores, Patricia Moreira-Escriche, Carmen De Juan, Salvador Resino, Amanda Fernández-Rodríguez, María Ángeles Jiménez-Sousa

**Affiliations:** ^1^Unit of Viral Infection and Immunity, National Center for Microbiology (CNM), Health Institute Carlos III (ISCIII), Madrid, Spain; ^2^Department of Infectious Diseases, Hospital Universitario Infanta Leonor, Madrid, Spain; ^3^Critical Care Department, Hospital Universitario del Tajo, Aranjuez, Spain; ^4^Clinical Microbiology Department, Hospital Universitario Príncipe de Asturias, Alcalá de Henares, Spain; ^5^Haematology and Haemostasis Department, University Hospital Infanta Leonor, Madrid, Spain; ^6^Unit AIDS Research Network Cohort (CoRIS), National Center of Epidemiology (CNE), Health Institute Carlos III (ISCIII), Madrid, Spain; ^7^Clinical Analysis Service, Hospital Universitario Príncipe de Asturias, Alcalá de Henares, Spain; ^8^Allergology Department, University Hospital Infanta Leonor, Madrid, Spain; ^9^Department of Infectious Diseases, Hospital Universitario Severo Ochoa, Madrid, Spain

**Keywords:** SARS-CoV-2, coagulation factors, COVID-19, mortality, SARS

## Abstract

**Background:** The link between coagulation system disorders and COVID-19 has not yet been fully elucidated.

**Aim:** Evaluating the association of non-previously reported coagulation proteins with COVID-19 severity and mortality.

**Design:** Cross-sectional study of 134 COVID-19 patients recruited at admission and classified according to the highest COVID-19 severity reached (asymptomatic/mild, moderate, or severe) and 16 healthy control individuals.

**Methods:** Coagulation proteins levels (antithrombin, prothrombin, factor_XI, factor_XII, and factor_XIII) and CRP were measured in plasma by the ProcartaPlex Panel (Invitrogen) multiplex immunoassay upon diagnosis.

**Results:** We found higher levels of antithrombin, prothrombin, factor XI, factor XII, and factor XIII in asymptomatic/mild and moderate COVID-19 patients compared to healthy individuals. Interestingly, decreased levels of antithrombin and factors XI, XII, and XIII were observed in those patients who eventually developed severe illness. Additionally, survival models showed us that patients with lower levels of these coagulation proteins had an increased risk of death.

**Conclusion:** COVID-19 provokes early increments of some specific coagulation proteins in most patients. However, lower levels of these proteins at diagnosis might “paradoxically” imply a higher risk of progression to severe disease and COVID-19-related mortality.

## How This Fits in

COVID-19 causes an early increase in coagulation proteins in most patients, even in those asymptomatic or with mild symptoms. Although not reflected in routine tests such as PT and aPTT, patients who lately advanced to severe disease, showed low levels of antithrombin, prothrombin, and factors XI, XII, and XIII at disease diagnosis. These reduced levels were associated for the first time with a higher COVID-19-related mortality.

## Introduction

Coronavirus disease 2019 (COVID-19) is associated with a significant activation of the coagulation cascade. While thrombosis has been classically described in acute and chronic infections including respiratory diseases (1), thrombotic risk appears to be higher in COVID-19 (2). Consequently, thromboembolic complications are common in hospitalized patients, especially among those in intensive care units (ICUs) (3). In this sense, several mechanisms of coagulation activation have been postulated (4) and large dynamic fluctuations in coagulation and fibrinolysis laboratory parameters have been described during disease course (5). Development of overt disseminated intravascular coagulation (DIC) seems to be rare and to follow a different pattern from other infection-derived DIC (5–7), but it has been reported in up to 71% of fatal cases as a late and ominous sign (8).

Additionally, both venous and arterial thrombotic events have been independently associated with mortality (9). Several hemostatic-system abnormalities such as thrombocytopenia, elevated D-dimer levels, prolonged prothrombin time (PT) or activated partial thromboplastin time (aPTT), decreased factor V activity, hypofibrinogenemia, and reduced levels of natural anticoagulants (e.g., antithrombin) appear with increasing disease severity and have been linked to death (6, 8, 10). However, the bidirectional relationship between SARS-CoV-2 and the coagulation system is still not completely understood (4). A predominant increase of D-dimer is typical, and its presence on admission has been repeatedly described as significantly higher in non-survivors (11) but scarce or no abnormalities in PT and aPTT are usually found at disease onset (5, 12). To date, coagulation markers measured in the early phase of COVID-19 have evidenced a complex scenario and elucidation of the pathophysiology of immunothrombosis is evolving. Therefore, to continue unraveling the insights of COVID-19-induced coagulopathy, we evaluate several coagulation proteins at an early stage of disease and their association with disease severity and mortality.

## Methods

### Design and Study Population

In this cross-sectional study, 128 COVID-19 non-selected patients consecutively admitted to three different hospitals in Madrid (Infanta Leonor University Hospital, Aranjuez University Hospital, and Príncipe de Asturias University Hospital), with an available plasma sample, were enrolled from March to September 2020.

**Figure 1:**
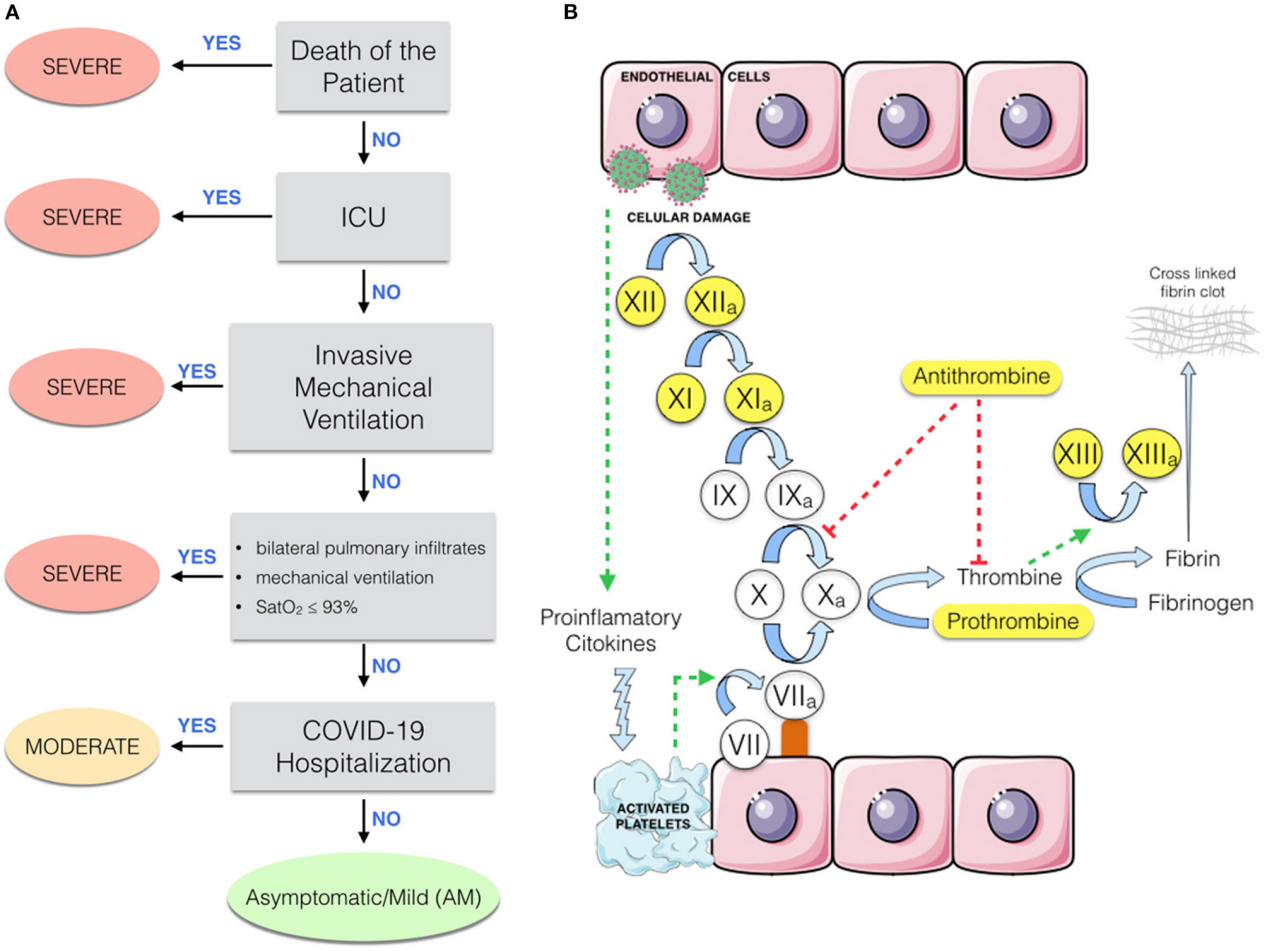
**(A)** Flowchart describing the classification of patients with respect to COVID-19 severity. ICU, intensive care unit; ARDS, acute respiratory distress syndrome; AM, asymptomatic/mild patients. **(B)** Partial representation of the coagulation cascade. Proteins analyzed by this study are highlighted in yellow.

Patients were classified according to their highest severity grade during the course of COVID-19 (Figure 1): (1) Severe: (i) death during hospitalization, (ii) ICU admission, (iii) invasive mechanical ventilation, or (iv) presence of bilateral pulmonary infiltrates, mechanical ventilation, and oxygen saturation (Sat0_2_) ≤93. (2) Moderate: the remaining hospitalized patients who did not fulfill the severe COVID-19 criteria; (3) asymptomatic/mild (AM): individuals who had minor or no COVID-19 symptoms; (4) a control group of 16 pre-pandemic healthy controls without any known infection was included, they were age- and sex-matched with COVID-19 groups to limit confounding factors. The STROBE-ID checklist was used to strength the design and conduct the study.

### Clinical Data and Samples

Epidemiological, clinical, and disease evolution data, as well as laboratory parameters such as PT, international normalized ratio (INR), and aPTT were collected from clinical records using an electronic case report form (eCRF) which was built using REDCap (13). Plasma samples were obtained after centrifugation of blood in EDTA tubes at hospital admission (median = 2 days, IQR = 4 days). Samples were processed at the National Center for Microbiology (Majadahonda), Institute of Health Carlos III (Madrid, Spain).

### Coagulation Proteins

Coagulation proteins (antithrombin, prothrombin, factor XI, factor XII, and factor XIII) and C-reactive protein (CRP) were measured by a Human Coagulation 6-PLEX ProcartaPlex Panel (Invitrogen), and D-dimer and IL-6 were measured by a multiplex immunoassay using a Bio-Plex 200™ system (Bio-Rad) according to the manufacturer's specifications. Coagulation proteins and their relationship in the coagulation cascade are shown in Figure 1.

### Statistical Analysis

As outcome variables, the highest COVID-19 severity and mortality were considered. For descriptive data, differences between groups were tested using Chi^2^ or Fisher's exact test with a Monte Carlo-simulated *p*-value for categorical variables and Kruskal-Wallis test for continuous variables. The association between coagulation proteins, measured in the first days of disease, and the severity were explored using generalized linear mixed models (GLMMs). This analysis allows us to test the pairwise difference among disease severity classes by grouping the four coagulation proteins analyzed in this study. We fit a model where the protein was considered a random effect:


(1) $$\begin{array}{l} \begin{array}{c} Y_{Severity\;class\;A,\;severity\;class\;B}=(\beta_{0}+b_{p,0p})+\beta_{1}X_{i} \end{array}\\ \begin{array}{c} \beta_{2}X_{j}+\beta_{3}X_{k}+e_{pijk} \end{array} \end{array}$$

Where bp, 0p is the random effect of each coagulation protein, β1Xi is the fixed effect of the protein levels, β2Xj is the fixed effect of the age, and β3Xk is the fixed effect of the sex. By using this model, we tested whether we were able to detect general effects between disease severity classes. We were also interested in studying the behavior of each coagulation protein. Pairwise comparisons between disease severity classes, for each protein, were carried out using multivariable logistic regressions. Sex and age were included as covariables in the multivariable analysis.

Survival time was defined as time between hospitalization and death, and individuals alive at 90 days were considered censored data. Survival curves were modeled using the Kaplan Meier method and a log rank test was performed to assess univariate differences in survival time according to coagulant proteins tertile levels. Death risk was estimated with the Cox proportional-hazard and Aalen's additive models. Age and sex were included as covariables. The survival Cox proportional-hazard model's goodness of fit was evaluated by the Harrel's concordance index (C-index). This index ranges from 0 to 1 and the intuition behind it is that, if the risk model is good, patients who had shorter times-to-death should have higher risk scores. Values of C-index near 0.5 indicate that the risk score predictions are not better than chance in determining which individual will die first. Coagulation proteins discrimination capabilities were measured by the area under the receiver operating characteristic (ROC) curve.

Two-sided tests were used for all statistical methods. Analyses were performed using the R 4.0.3 software.

## Results

### Clinical and Epidemiological Data of Patients

Patient's characteristics are shown in Table 1. Coagulation proteins were measured before therapy administration, and no associations were found with comorbidities, chronic medications, or COVID-19 presentation (Supplementary Table 1; Supplementary Figure 1), showing no prior bias. Additionally, aPTT and PT (INR) did not show differences among the three groups of COVID-19 patients (Supplementary Figure 2).

**Table 1:** Patient's characteristics.

	**COVID-19 severity**			
	**Healthy**	**Asymptomatic/mild**	**Moderate**	**Severe**
Demographics				
N	16	13	68	47
Age	58.8 ± 12.3	64.3 ± 18.1	61.1 ± 15.3	62.1 ± 18.1
Gender (male)	9/16 (56.2%)	5/13 (38.4%)	38/68 (55.8%)	33/47 (70.2%)
BMI ≥ 25	8/11 (72.7%)	2/12 (16.6%)	11/68 (16.1%)	13/47 (27.6%)
Smoke status (Yes)	NA	2/13 (15.3%)	3/68 (4.4%)	3/47 (6.3%)
Former smoker	NA	4/13 (30.7%)	8/68 (11.7%)	11/47 (23.4%)
Comorbidities				
Hypertension	NA	5/13 (38.4%)	30/68 (44.1%)	23/47 (48.9%)
Cardiopathy	NA	4/13 (30.7%)	12/68 (17.6)	8/47 (17.0%)
Chronic pulmonary disease	NA	0/13 (0%)	8/68 (11.7%)	10/47 (21.2%)
Chronic kidney disease	NA	2/13 (15.3%)	4/68 (5.8%)	9/47 (19.1%)
Chronic liver disease	NA	NA	4/65 (6.2%)	2/47 (4.2%)
Chronic neurological disease	NA	0/13 (0%)	9/67 (13.4%)	8/47 (17.0%)
Neoplasia	NA	NA	3/63 (4.8%)	4/47 (8.5%)
Diabetes	NA	2/13 (15.3%)	10/68 (14.7%)	11/47 (23.4%)
Chronic inflammatory disease	NA	NA	2/63 (3.1%)	5/47 (10.6%)
Autoimmune disease	NA	NA	1/63 (1.5%)	4/47 (8.5%)
Therapy				
Chronic medications				
NSAIDs	NA	0/13 (0%)	0/68 (0%)	7/47 (14.9%)
ACE inhibitors	NA	0/13 (0%)	15/68 (22.3%)	11/47 (23.4%)
ARBs	NA	1/13 (7.6%)	7/68 (10.4%)	8/47 (17.0%)
Corticosteroids	NA	NA	6/68 (9.5%)	6/47 (12.8%)
HIV antiretrovirals	NA	NA	1/68 (1.5%)	1/47 (2.1%)
Treatment				
Chloroquine and Hydroxychloroquine	0/16 (0%)	0/13 (0%)	62/63 (98.4%)	33/47 (70.2%)
Tocilizumab	0/16 (0%)	0/13 (0%)	10/67 (15.0%)	20/47 (42.5%)
Corticosteroids	0/16 (0%)	0/13 (0%)	26/67 (38.8%)	40/47 (85.1%)
COVID-19 related symptoms				
Dyspnea	0/16 (0%)	0/13 (0%)	43/68 (63.2%)	41/47 (87.2%)
Cough	0/16 (0%)	0/13 (0%)	48/63 (76.1%)	34/47 (72.3%)
Headache	0/16 (0%)	0/13 (0%)	24/63 (38.1%)	10/47 (21.2%)
Diarrhea or abdominal pain	0/16 (0%)	0/13 (0%)	33/63 (52.4%)	19/47 (40.4%)
Hospitalization				
Length of stay (days)	0/16 (0%)	0/13 (0%)	10.7 ± 8.6	22.1 ± 15.6
Maximum temperature	NA	NA	37.8 ± 0.8	38.2 ± 0.8
Oxygen therapy	0/16 (0%)	0/13 (0%)	39/68 (57.3%)	47/47 (100%)
Non-invasive mechanical ventilation	0/16 (0%)	0/13 (0%)	3/67 (4.5%)	20/47 (38.99%)
Invasive mechanical ventilation	0/16 (0%)	0/13 (0%)	0/67 (0%)	18/47 (38.2%)
Pulmonary infiltrates	0/16 (0%)	0/13 (0%)	60/68 (88.2%)	47/47 (100%)
ICU				
ICU length of stay	0	0	0	6.61 ± 13.5
Death	0/16 (0%)	0/13 (0%)	0/68 (0%)	18/47 (38.3%)

*Patients were classified according to the highest disease severity reached during the COVID-19 evolution. Denominator indicates number of patients with available data. **Statistics**: Individual characteristics were summarized using standard descriptive statistics: mean ± standard deviation for continue variables and count (percentage) for categorical variables. Differences between groups were tested using Fisher's exact test. No statistically significant association was found between covariates and disease severity with the exception of the following treatments: Chloroquine/hydroxychloroquine (Fisher's exact test = 1.6e^−05^), Tocilizumab (Fisher's exact test = 8.1e^−05^), corticosteroids (Fisher's exact test = 5.7e^−08^), and the use of supplemental oxygen (Fisher's exact test = 3.2.e^−07^). NA, non-available; BMI, body mass index; NSAIDs, non-steroidal anti-inflammatory drugs; ACE, angiotensin-converting-enzyme; ARBs, angiotensin II inhibitors; HIV, human immunodeficiency virus; ICU, intensive care unit*.

### Coagulation Proteins Association With Severity

**Figure 2:**
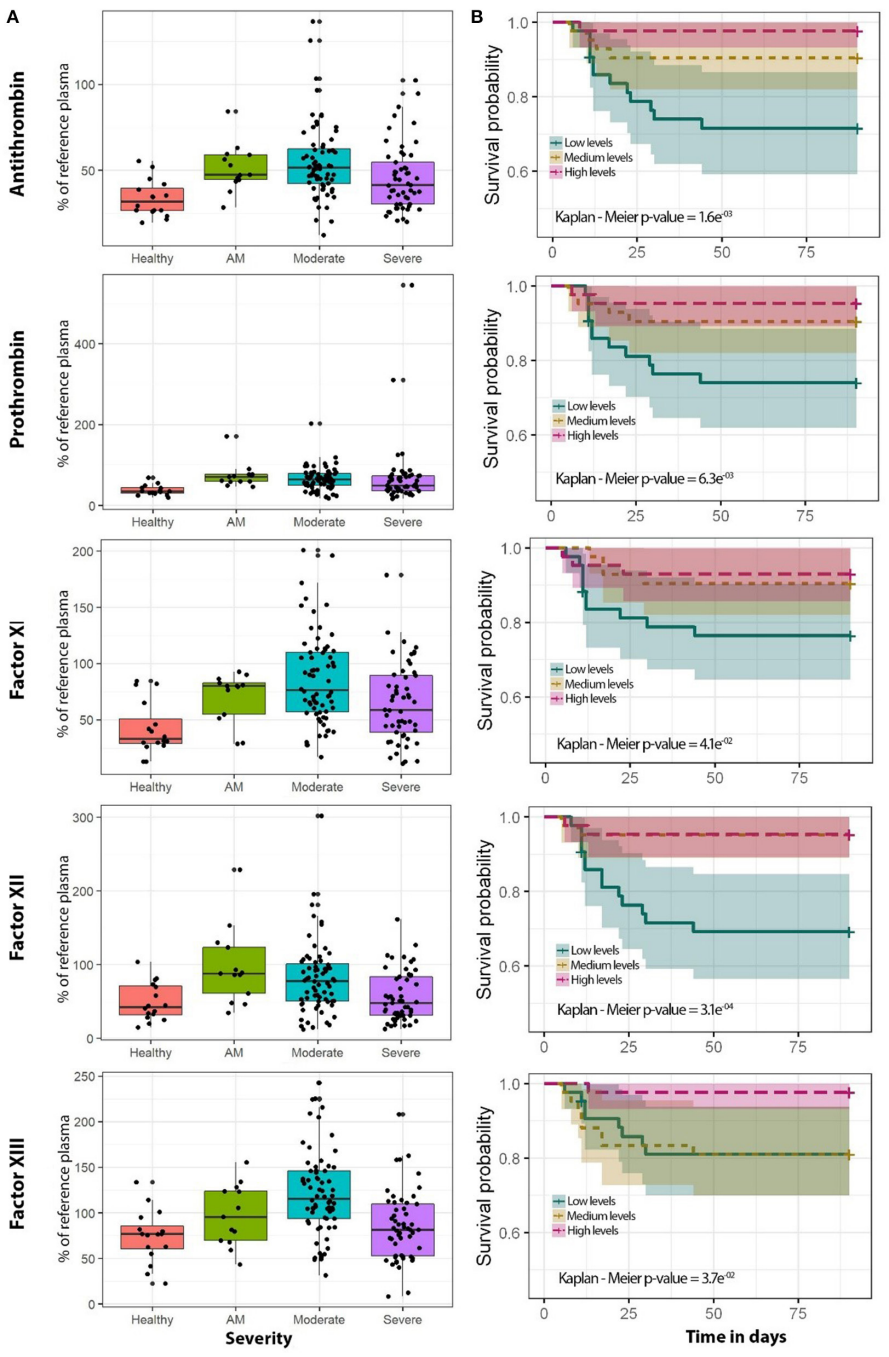
Coagulation protein levels regarding COVID-19 severity and survival analysis for each of the coagulation proteins analyzed in this study. **(A)** Boxplots of healthy (purple, *n* = 16), AM (blue, *n* = 13), moderate (green, *n* = 68), and severe (yellow, *n* = 47) individuals. **(B)** Kaplan-Meier plot. The cutoffs of coagulation proteins for the Kaplan-Meier plot were obtained using three quantiles to get low (blue), medium (yellow), and high (pink) percentages of the reference plasma. The specific categorical levels by using tertiles were as follows: Antithrombin: low (12.14–41.3), medium (41.3–54.1), high (54.1–136.7). Prothrombin: low (16.3–48.7), medium (48.7–72.2), high (72.2–545). Factor XI: low (10.8–56.4), medium (56.4–90.1), high (90.1–200.9). Factor XII: low (11.7–47.8), medium (47.8–87.1), high (87.1–301.9). Factor XIII: low (8.3–82.6), medium (82.6–118.3), high (118.3–242.7). AM, asymptomatic/mild patients; OLR, ordinal logistic regression.

**Figure 3:**
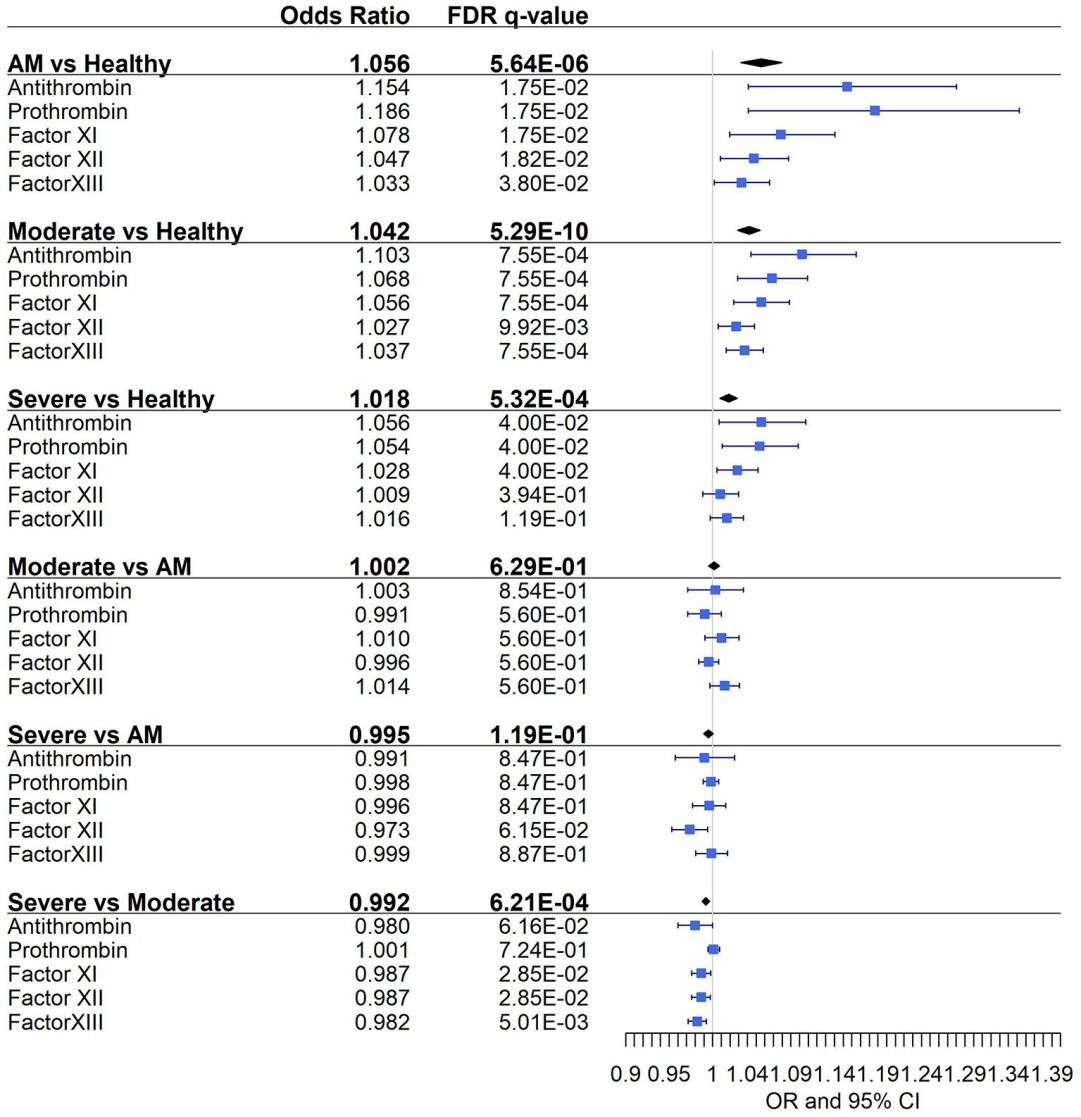
Pairwise association between coagulation protein levels and the different severity classes. Statistics: Pairwise comparisons between COVID-19 severity classes were obtained using a logistic mixed model where the protein was considered a random effect. Pairwise comparisons within each protein were obtained using multiple logistic regression analysis. The False Discovery Rate or FDR was used to cope with multiple testing, q-values are provided. AM, asymptomatic/mild patients.

Distribution of antithrombin, prothrombin, factor XI, factor XII, and factor XIII protein levels for each disease severity group is shown in Figure 2A. Statistical differences between severity groups are shown through generalized linear models in Figure 3. AM, moderate, and severe individuals had higher levels of coagulation proteins compared to healthy individuals. Also, we detected a significant reduction of factor XI, factor XII, and factor XIII levels in severe patients compared to moderate individuals (Supplementary Table 2). These pairwise differences between COVID-19 severity groups were also detected at the individual protein level (Figure 3; Supplementary Table 3).

Additionally, a traditional coagulation activation marker such as D-dimer and surrogate markers of inflammation such as CRP and IL-6 were also assessed. Statistical differences were observed mainly between moderate and severe groups, except for CRP, for which extreme groups showed the highest differences (Supplementary Table 4; Supplementary Figure 3). D-dimer showed significant correlation with prothrombin (*R*^2^ = 0.698, *P* = 2.1E^−22^) and factor XIII (*R*^2^ = −0.216, *P* = 3.7E^−03^).

### Survival Analysis

Kaplan-Meier analyses showed that patients with lower levels of the analyzed proteins had an increased risk of death during hospitalization (Figure 2B). For antithrombin, prothrombin, and factors XI and XIII, this increase was more accentuated in men (Supplementary Figure 4). The effect of proteins levels on survival was confirmed by the Cox proportional-hazard models and Aalen's additive regression (Supplementary Table 5). Both models found a significant effect of the different coagulation proteins on survival. These models found a negative correlation between the proteins' levels and the time of survival (Supplementary Table 5). A reduction in the coagulation proteins levels involves a higher risk of death, however, the effect size was slight for each individual protein (Supplementary Table 5). ROC curves showed that the addition of the coagulation proteins to the basic model composed by sex and age slightly improved the survival prediction although it was not significant (Supplementary Figure 5). We also performed survival analysis with aPTT and INR using different statistical models, but no significant association was observed (data not shown).

## Discussion

Our study shows that coagulation protein levels are affected at the first stages of COVID-19 and that these early changes already reflect disease severity. We report increased levels of antithrombin, prothrombin, factor XI, factor XII, and factor XIII in AM and moderate patients, compared to healthy individuals. In contrast, significantly decreased levels of antithrombin and factors XI, XII, and XIII were found at presentation in patients who followed a severe course with respect to moderate patients. By considering the coagulation protein as a random effect (in a logistic mixed model context), we could suggest a general activation of the coagulation cascade between healthy individuals and COVID-19 patients, and also a general decrease in protein levels in severe COVID-19 patients. As expected, D-dimer and IL-6 were significantly higher in severe patients than moderate patients. Moreover, CRP showed higher levels in moderate and severe patients than healthy and AM individuals. Additionally, aPTT and PT (INR) did not show differences among the three groups of COVID-19 severity, neither with survival. When we explored the effects of the coagulant proteins' levels on survival defined as time-to-death, we observed that patients with lower levels of the analyzed proteins had an increased risk of death during hospitalization.

Finally, we describe a sex-specific effect in further analyses, showing differences in the coagulation cascade regulation in both men and women. Variable sex was significant in the general mixed model and survival analysis. The negative association between coagulation proteins and disease severity was, in general, more pronounced in men. Our results seem to be in agreement with the clinical experience that found that men hospitalized with COVID-19 had more severe thrombosis than women (14).

### Strength and Limitations

We present the evaluation of coagulation factors underrepresented in the literature as an additional approach to study coagulopathy in COVID-19 disease. In the results we report new associations of some protein factors with COVID-19 severity and include specific groups of patients with scarce coagulation data, such as asymptomatic patients. We also add healthy non-hospitalized individuals to address for collider bias (15). Furthermore, we were able to detect a sex-specific effect of SARS-CoV-2 on coagulation protein levels. Two main limitations should be considered. First, the limited sample size of healthy and asymptomatic cases, which could have limited the possibility of finding statistically significant differences for some comparisons. Secondly, we analyzed five coagulation proteins not routinely used in clinical practice; as coagulation cascade is extremely complex, further studies should consider additional factors to fully describe COVID-19 effects over the entire coagulation cascade. However, these additional factors have been extensively studied, and we analyzed those that were not previously addressed.

### Comparison With Existing Literature

Since its worldwide outbreak in the first trimester of 2020, it has been systematically reported that COVID-19 is associated with a significant activation of the coagulation cascade. Our results are in accordance with previous studies (16) that show a consumption of coagulation proteins among COVID-19 non-survivors, or a reduction in abundance of prothrombin correlated with disease severity (17). Several recently published meta-analyses and reviews have shown significantly higher levels of D-dimer, fibrinogen, and fibrin in severe COVID-19 patients in comparison to non-severe (18–23).

However, as far as we know this is the first report studying coagulation factors XI, XII, and XIII in COVID-19, as most of the published studies have focused in other parameters such as platelets, D-dimer, fibrin, fibrinogen, aPTT, PT, and even factor VIII and thrombin (18, 22, 23).

The increase in natural anticoagulant and procoagulant proteins in COVID-19 has been attributed to the thromboinflammatory response caused by SARS-CoV-2, which provokes endotheliitis and increases the hepatic production of factors (24). This could explain the increases of clotting proteins found in our study even in early and mild stages.

Additionally, we showed that this negative association was more pronounced in men.

We observed that at the time of admission, there were no differences in aPTT and TP between survivors or non-survivors. These results are also in line with previous studies (2), where normal to slightly elevated aPTT or PT have been described in most COVID-19 patients at presentation. The INR, measured at the acute phase of the disease, seems to be elevated in non-survivors compared to survivors (8), however, no correlation was evident between COVID-19 severity and other DIC indicators like prolonged aPTT (16). Therefore, we suggest that an early reduced production or, more likely, an increased consumption (due to pulmonary or systemic coagulopathy) of clotting proteins could predict a worse prognosis.

The survival results were in accordance with previous studies (15) that show a consumption of coagulation proteins among COVID-19 non-survivors, however, we cannot rule out an impaired production of these coagulation proteins.

### Implications for Research and/or Practice

In conclusion, our results indicate that: (1) COVID-19 causes an early increase of some specific coagulation proteins such as antithrombin, prothrombin, contact factors, and factor XIII in most patients, even in those who will not suffer from clinically significant disease, suggesting that commonly elevated D-dimer levels are driven by an initial enhanced procoagulant state and not just by hyperfibrinolysis; (2) although not reflected in routine tests such as PT and aPTT, and despite common initial hyperfibrinogenemia, patients who will eventually advance to severe disease show early decreased levels of these anticoagulant and procoagulant markers, suggesting either consumption or impaired production, and these levels were associated with higher COVID-19-related mortality. Evolving investigations will allow us to better clarify the crosstalk between the immune and clotting systems in this pandemic disease.

## Data Availability Statement

The raw data supporting the conclusions of this article will be made available by the authors, without undue reservation.

## Ethics Statement

The studies involving human participants were reviewed and approved by the Ethics Committee of the Institute of Health Carlos III (PI 33_2020-v3) and the Ethics Committee of each hospital. The patients/participants provided their written informed consent to participate in this study.

## Author Contributions

AF-R and MJ-S: funding body, supervision, and visualization. AF-R, MJ-S, and FC: study concept and design, statistical analysis, interpretation of data, and writing of the manuscript. PR, RB, MM-V, AV-B, OB-K, FC, FP-G, OM-G, CV-M, NB-L, and IR: patients' selection and clinical data acquisition. MM-V, AV-B, EV-A, SB, and OB-K: sample preparation and biomarker analysis. FC, AF-R, and MJ-S: writing of the manuscript. PR, RB, FP-G, JC-S, OM-G, MR, PM-E, CD, and SR: critical revision of the manuscript for relevant intellectual content. All authors read and approved the final manuscript.

## Funding

This study was supported by grants from Instituto de Salud Carlos III [ISCIII; Grant Number COV20/1144 (MPY224/20) to AF-R/MJ-S]. AF-R, MJ-S, and MR are Miguel Servet researchers supported and funded by ISCIII (Grant Numbers: CP14CIII/00010 to AF-R, CP17CIII/00007 to MJ-S, and CP19CIII/00002 to MR).

## Conflict of Interest

The authors declare that the research was conducted in the absence of any commercial or financial relationships that could be construed as a potential conflict of interest.

## Publisher's Note

All claims expressed in this article are solely those of the authors and do not necessarily represent those of their affiliated organizations, or those of the publisher, the editors and the reviewers. Any product that may be evaluated in this article, or claim that may be made by its manufacturer, is not guaranteed or endorsed by the publisher.
