# Supplementary material for: Are Reduced Levels of Coagulation Proteins Upon Admission Linked to COVID-19 Severity and Mortality?
Source: Front Med (Lausanne). 2021 Sep 30;8:718053. doi: 10.3389/fmed.2021.718053 (PMC8514618; doi:10.3389/fmed.2021.718053)
Supplement: Supplementary file 1 [file Data_Sheet_1.PDF]

SUPPLEMENTARY MATERIAL AND METHODS.

Table S1. Differences in the levels of coagulation proteins regarding discrete covariables.

	Antithrombin	Prothrombin	Factor_XI	Factor_XII	Factor_XIII
Sex	0.996	0.933	0.933	0.971	0.623
BMI>=25	0.832	0.683	0.683	0.799	0.623
Smoker	0.600	0.323	0.364	0.517	0.314
Hypertension	0.832	0.683	0.683	0.872	0.623
Cardiopathy	0.832	0.933	0.933	0.799	0.658
Chronic pulmonary disease	0.832	0.323	0.364	0.517	0.917
Chronic kidney disease	0.832	0.323	0.364	0.799	0.314
Chronic liver disease	0.947	0.683	0.683	0.872	0.658
Chronic neurological disease	0.832	0.933	0.933	0.872	0.623
Neoplasia	0.832	0.683	0.683	0.872	0.624
Diabetes	0.956	0.933	0.933	0.948	0.314
Chronic inflammatory disease	0.625	0.933	0.933	0.872	0.413
Autoimmune disease	0.996	0.933	0.933	0.872	0.643
NSAIDs	0.947	0.718	0.718	0.958	0.658
ACE inhibitors	0.947	0.683	0.683	0.932	0.658
ARBs	0.947	0.683	0.683	0.932	0.623
Dyspnoea	0.996	0.683	0.683	0.932	0.658
Cough	0.996	0.683	0.683	0.981	0.658
Headache	0.915	0.946	0.946	0.902	0.314
Diarrhea or abdominal pain	0.600	0.323	0.364	0.517	0.717

Statistics: Differences were evaluated by Kruskal Wallis rank sum test (False Discovery Rate (FDR) q-values for the Kruskal Wallis are shown)**. Abbreviations**: BMI, body mass index; NSAIDs, Nonsteroidal anti-inflammatory drugs; ACE, Angiotensin-converting-enzyme; ARBs, angiotensin II inhibitors.

Table S2. Pairwise differences between COVID-19 severity classes.

	OR	2.5%	97.5%	FDR q-value
**AM vs Healthy**				
Prot. levels	1.056	1.033	1.080	< 0.001
Age	0.946	0.918	0.976	< 0.001
Sex	0.289	0.119	0.700	0.005
**Moderate vs Healthy**				
Prot. levels	1.042	1.029	1.055	< 0.001
Age	0.983	0.965	1.001	0.071
Sex	1.222	0.705	2.119	0.476
**Severe vs Healthy**				
Prot. levels	1.018	1.008	1.028	< 0.001
Age	1.004	0.987	1.021	0.674
Sex	2.428	1.420	4.151	0.001
**Moderate vs AM**				
Prot. levels	1.002	0.995	1.008	0.629
Age	1.021	1.005	1.037	0.010
Sex	3.181	1.785	5.670	< 0.001
**Severe vs AM**				
Prot. levels	0.995	0.990	1.001	0.119
Age	1.018	1.002	1.034	0.027
Sex	5.297	2.889	9.715	< 0.001
**Severe vs Moderate**				
Prot. levels	0.992	0.988	0.997	< 0.001
Age	1.010	0.999	1.021	0.069
Sex	1.918	1.335	2.755	< 0.001

Statistics: A general linear mixed model (GLMM) was fit considering the coagulation protein a random effect. Protein concentration, age and sex were considered fixed effects. Odds ratio, 95% confidence intervals (lower boundary: 2.5%, upper boundary: 97.5%), and the False Discovery Rate (FDR) q-values are shown for every model.

Table S3. Association between coagulation proteins and COVID-19 severity.

	OR	2.5%	97.5%	FDR q-value
**AM vs Healthy**				
Antithrombin	1.155	1.041	1.280	0.017
Prothrombin	1.187	1.041	1.353	0.017
Factor XI	1.078	1.019	1.141	0.017
Factor XII	1.047	1.009	1.087	0.018
Factor XIII	1.033	1.002	1.065	0.038
**Moderate vs Healthy**				
Antitrombin	1.103	1.044	1.165	< 0.001
Prothrombin	1.068	1.029	1.109	< 0.001
Factor XI	1.056	1.024	1.088	< 0.001
Factor XII	1.027	1.006	1.048	0.009
Factor XIII	1.037	1.016	1.058	< 0.001
**Severe vs Healthy**				
Antitrombin	1.056	1.007	1.107	0.040
Prothrombin	1.054	1.011	1.099	0.040
Factor XI	1.028	1.005	1.052	0.040
Factor XII	1.009	0.989	1.030	0.394
Factor XIII	1.016	0.997	1.036	0.192
**Moderate vs AM**				
Antitrombin	1.003	0.971	1.036	0.854
Prothrombin	0.991	0.972	1.010	0.560
Factor XI	1.010	0.991	1.030	0.560
Factor XII	0.996	0.984	1.007	0.560
Factor XIII	1.014	0.997	1.031	0.560
**Severe vs AM**				
Antitrombin	0.991	0.957	1.025	0.847
Prothrombin	0.998	0.989	1.007	0.847
Factor XI	0.996	0.977	1.015	0.847
Factor XII	0.973	0.953	0.994	0.061
Factor XIII	0.999	0.981	1.017	0.887
**Severe vs Moderate**				
Antitrombin	0.980	0.960	1.000	0.061
Prothrombin	1.001	0.995	1.008	0.724
Factor XI	0.987	0.976	0.998	0.028
Factor XII	0.987	0.976	0.997	0.028
Factor XIII	0.982	0.972	0.993	0.005

Statistics: Pairwise logistic regression models were fit for each protein and pair of COVID-19 severity classes. Age and sex were added in each model as covariables. Odds ratio, 95% confidence intervals (lower boundary: 2.5%, upper boundary: 97.5%), and the False Discovery Rate (FDR) q-values are shown for every model.

Table S4. Association between D-dimer, CRP and IL-6, with COVID-19 severity.

	Log Odds	SD	P-value
**AM vs Healthy**			
D-dimer	1.58E-05	1.1E-05	0.1465
CRP	1.84E-04	1.8E-04	0.3249
IL-6	-0.0339	0.0365	0.3538
**Moderate vs Healthy**			
D-dimer	8.29E-06	8.44E-06	0.3255
CRP	5.87E-04	1.9E-04	**0.0013**
IL-6	-0.0159	0.013	0.2496
**Severe vs Healthy**			
D-dimer	1.94E-05	1.2E-05	0.1149
CRP	8.6E-03	2.1E-04	**<0.0001**
IL-6	0.013	0.0109	0.2342
**Moderate vs AM**			
D-dimer	-6.69E-06	5.95E-06	0.2598
CRP	3.79E-04	1.5E-05	**0.0012**
IL-6	0.0371	0.0453	0.4145
**Severe vs AM**			
D-dimer	2.62E-06	4.8E-06	0.5882
CRP	6.7E-04	1.9E-05	**<0.0001**
IL-6	0.0895	0.0472	0.0578
**Severe vs Moderate**			
D-dimer	7.45E-06	3.1E-07	**0.0179**
CRP	4.79E-06	3.7E-06	0.1961
IL-6	0.0378	0.0112	**0.0033**

Statistics: Pairwise logistic regression models were fit for each protein and pair of COVID-19 severity classes. Age and sex were added in each model as covariables. Log odds ratio, standard deviation, and p-values are shown for every model.

Table S5. Survival analysis in COVID-19 patients according to coagulation proteins´ levels.

	Cox Proportional-Hazard			Aalen's Additive Regression	
	**HR**	**p-value**	**C-index**	**Coefficient**	**p-value**
**Antithrombin**			0.883 ±0.033		
Antithrombin	0.962	0.020		-2.59E-03	0.001
Age	1.034	< 0.001		6.54E-04	< 0.001
Sex	3.191	0.056		9.33E-02	0.028
**Prothrombin**			0.862 ±0.048		
Prothrombin	1.006	0.055		5.61E-05	0.054
Age	1.121	< 0.001		6.31E-04	< 0.001
Sex	4.571	0.016		8.97E-03	0.033
**Factor_XI**			0.859 ±0.042		
Factor_XI	0.990	0.294		-1.25E-04	0.056
Age	1.108	< 0.001		6.66E-04	< 0.001
Sex	3.829	0.002		9.68E-03	0.024
**Factor_XII**			0.875 ±0.040		
Factor_XII	0.983	0.034		-8.43E-05	0.009
Age	1.097	< 0.001		6.12E-04	< 0.001
Sex	3.201	0.0501		8.41E-03	0.047
**Factor_XIII**			0.881 ±0.042		
Factor_XIII	0.980	0.033		-2.29E-03	0.001
Age	1.111	< 0.001		6.79E-04	< 0.001
Sex	3.017	0.0721		7.95E-03	0.050

Statistics: Cox Proportional-Hazard and Aalen's Additive Regression. Hazard ratio, p-values and Harrell's concordance index (C-index) are shown for the Cox models. C-index is a goodness of fit measure for models that produces risk scores. Models with higher C-index indicate a shorter time-to-disease for those patients with higher risk score. A C-index's value of 0.5 entails that the risk score predictions are no better than chance. Values near 1 indicates perfect separation of patients with different outcomes. Regression coefficients and their associated p-values are also shown for the Aalen's models. The Aalen model allows for time-varying covariate effects. Regarding the covariables it is possible to notice that age and sex have a positive and significant effect over mortality. Abbreviations: HR, hazard ratio.

Figure S1. Correlation between coagulation proteins and continuous covariables related to severity.

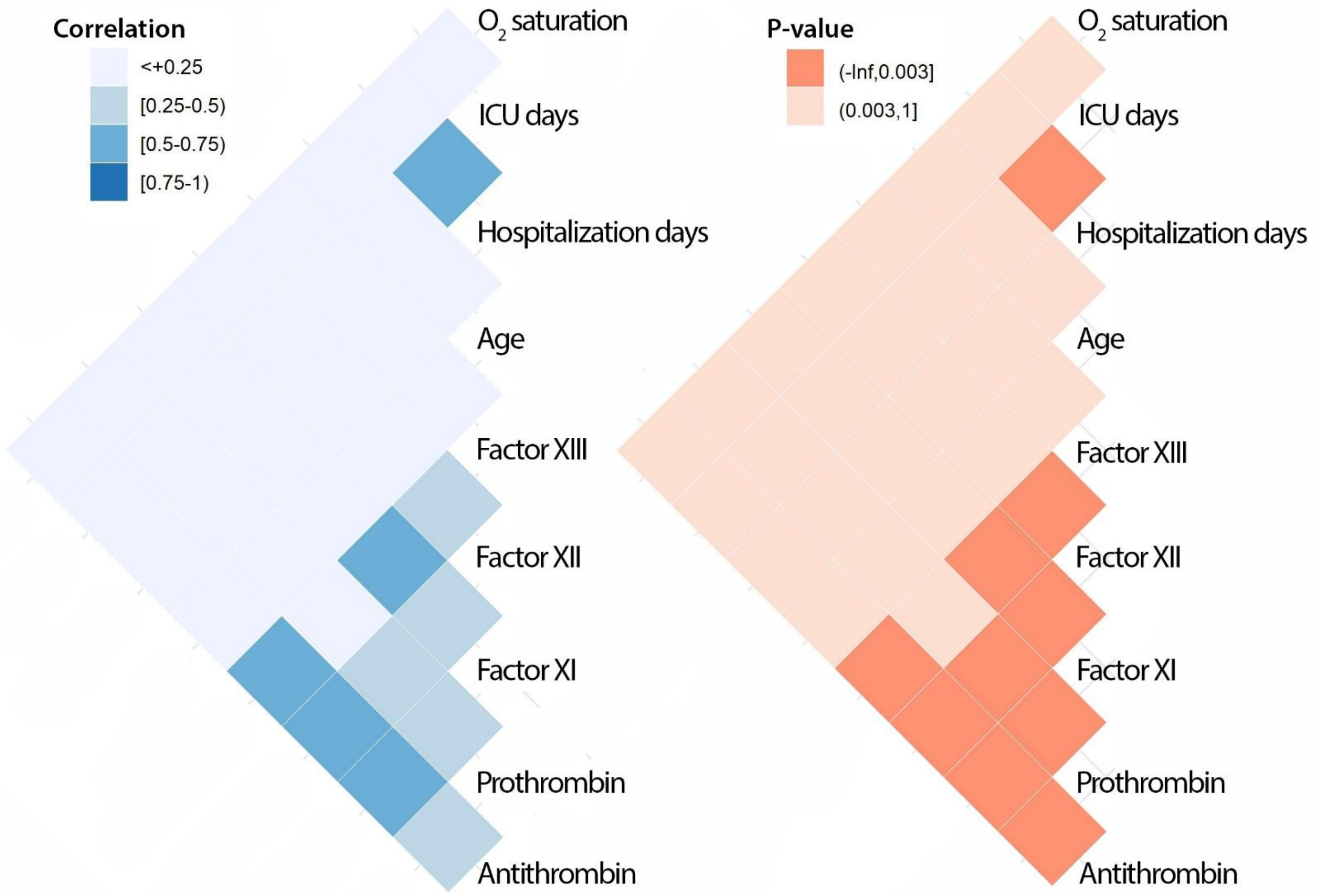

Statistics: Pearson correlation. Heatmaps are provided for the correlation coefficients and its associated p-values. A regular Bonferroni correction for multiple testing was applied, being significance set at $3.0e^{-03}$. **Abbreviations**: O_2, oxygen; ICU, intensive care unit.

Figure S2. Differences in activated partial thromboplastin time (APTT) and international normalized ratio (INR) between the severity groups.

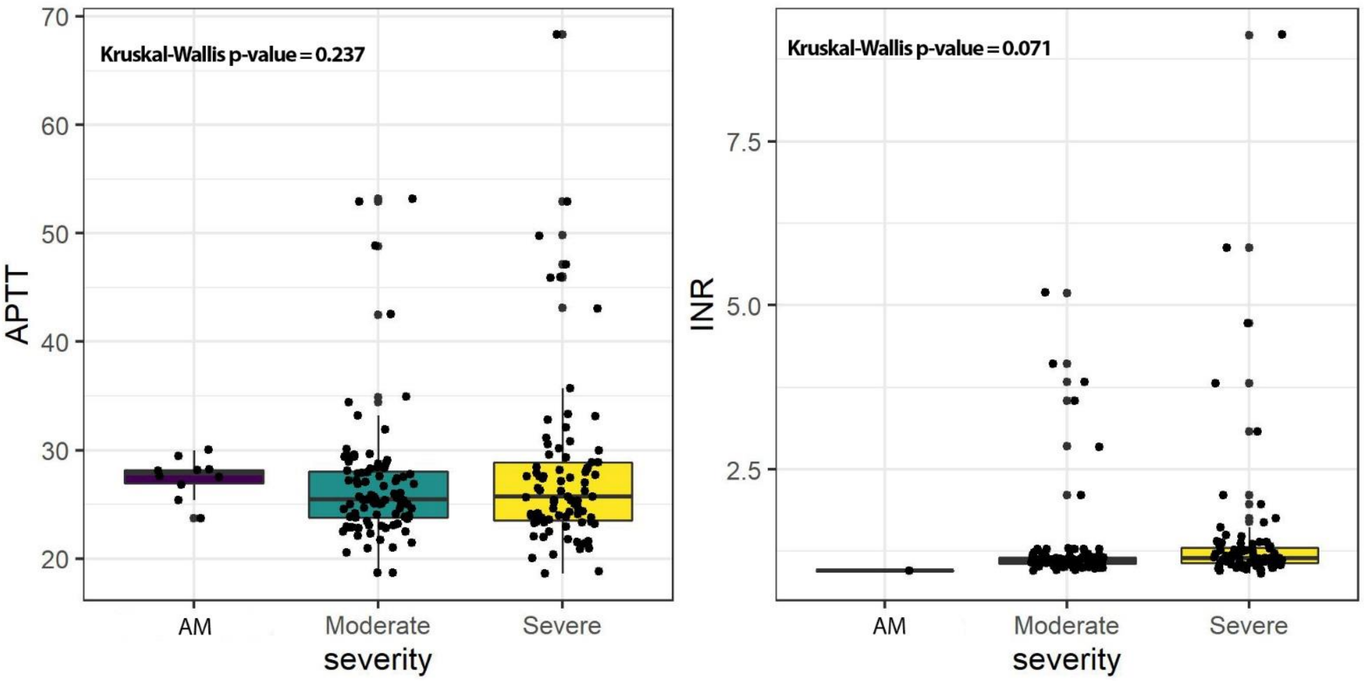

Statistics: Distributions were presented in box plots and significance was calculated by Kruskal-Wallis test. APTT and INR levels did not show differences among the three groups of COVID-19 (p-value = 0.237 and p-value = 0.071, respectively). **Abbreviations**: AM, asymptomatic/mild patients; APTT, activated partial thromboplastin time; INR, international normalized ratio.

Figure S3 Differences in D-dimer, C-reactive protein (CRP) and IL-6 between the severity groups.

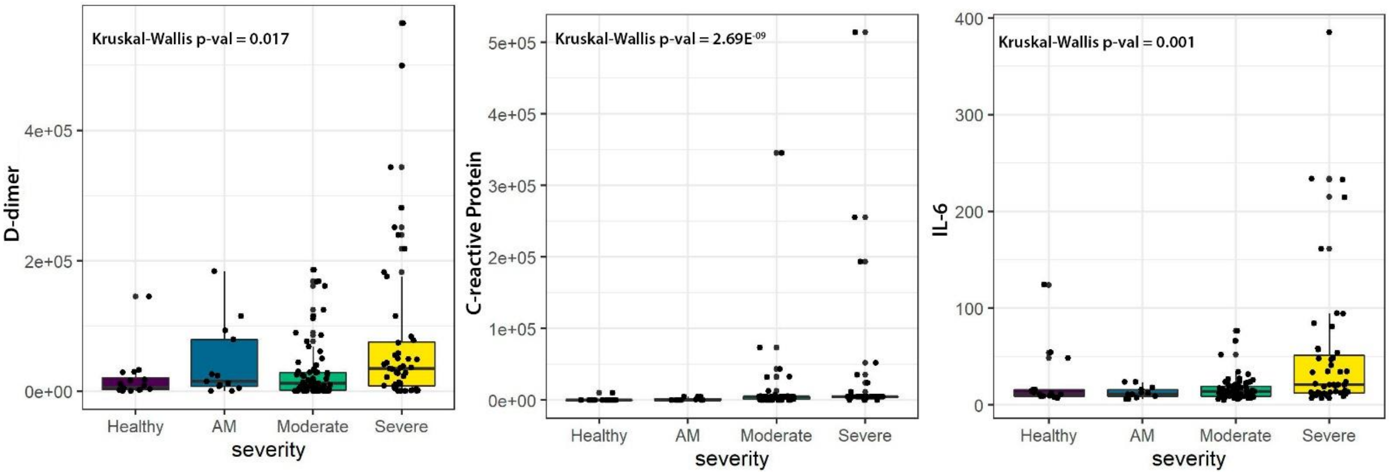

Statistics: Distributions were presented in box plots and significance was calculated by Kruskal-Wallis test. We find significant differences among the three groups of COVID-19 for the three biomarkers. **Abbreviations**: AM, asymptomatic/mild patients; APTT, activated partial thromboplastin time; INR, international normalized ratio.

Figure S4. **Kaplan-Meier plots regarding coagulation protein levels and grouped by sex**.

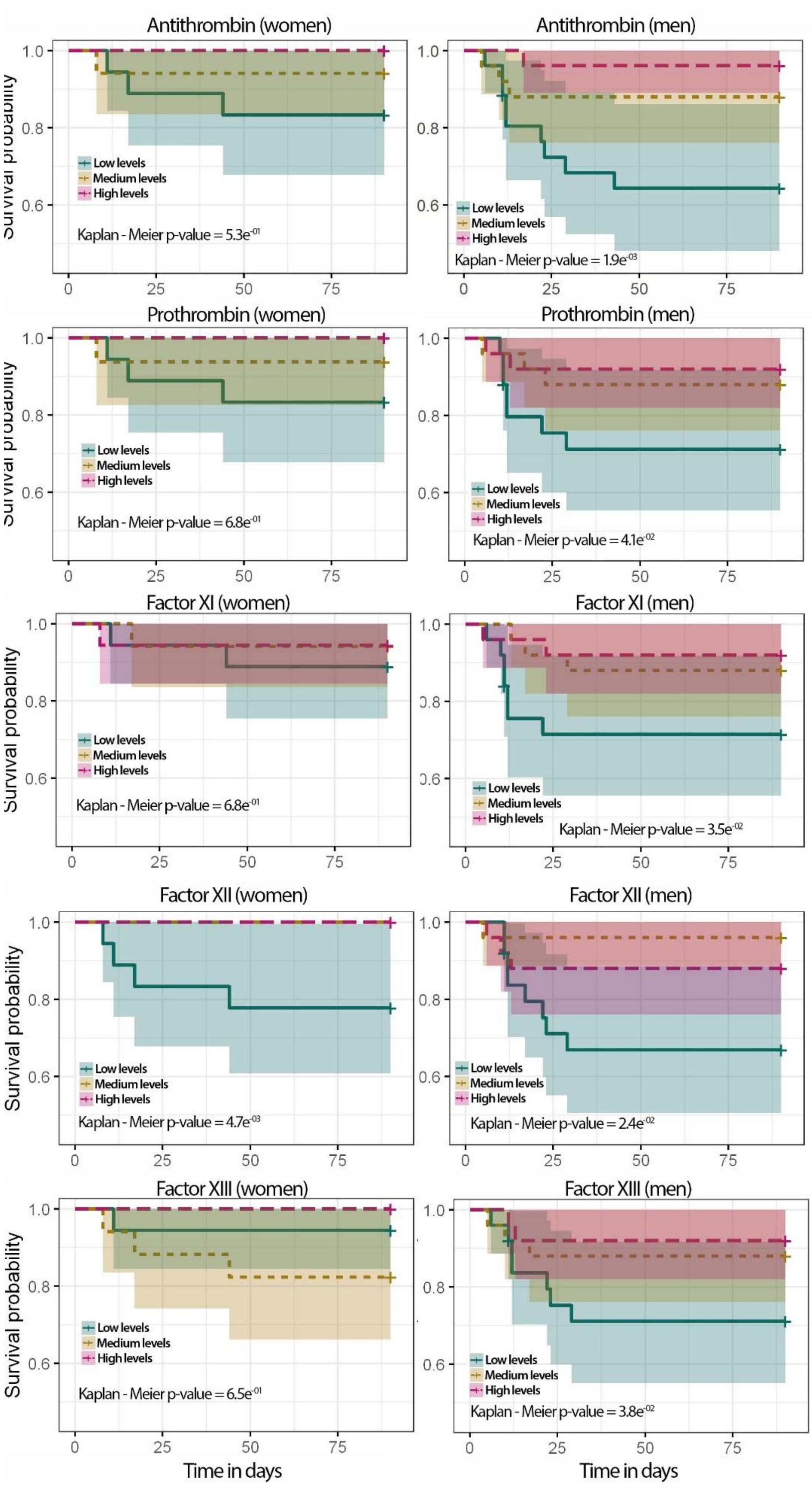

Statistics: Coagulation proteins´ classes were obtained using 3 quantiles to get low (blue), medium (yellow) and high (pink) factor levels. P-values of the Kaplan-Meier analysis are shown in each plot. Men = 75, Women = 53.

Figure S5. Predictive accuracy of the model with coagulation proteins in combination with epidemiological variables.

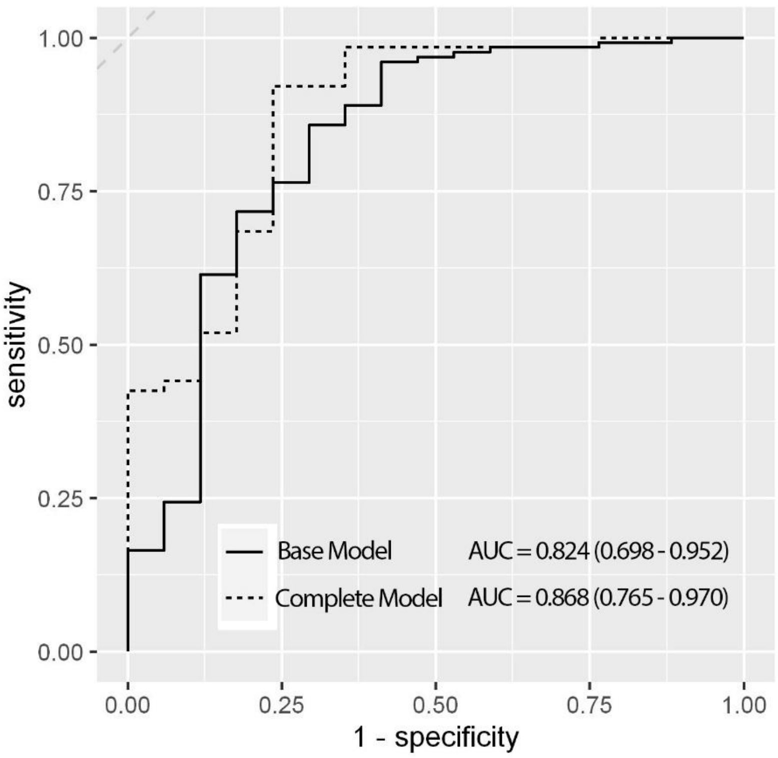

Statistics: ROC curves and the Area Under the Curve (AUC) with its 95% CI in brackets. Base model: mortality is modelled according to age and sex covariables. Complete Model: all five coagulation protein levels were included (antithrombin, prothrombin, Factor XI, Factor XII, Factor XIII) to the base model. No significant differences were found
